# Functional fibrillar interfaces: Biological hair as inspiration across scales

**DOI:** 10.3762/bjnano.15.55

**Published:** 2024-06-06

**Authors:** Guillermo J Amador, Brett Klaassen van Oorschot, Caiying Liao, Jianing Wu, Da Wei

**Affiliations:** 1 Experimental Zoology Group, Department of Animal Sciences, Wageningen University & Research, De Elst 1, 6708 WD Wageningen, Netherlands; 2 School of Aeronautics and Astronautics, Shenzhen Campus of Sun Yat-sen University, Shenzhen, 518107, China; 3 Beijing National Laboratory for Condensed Matter Physics and Laboratory of Soft Matter Physics, Institute of Physics, Chinese Academy of Sciences, Beijing 100190, China

**Keywords:** adhesion, fibers, fluid–structure interactions, mastigonemes, mechanosensing, setae, wettability

## Abstract

Hair, or hair-like fibrillar structures, are ubiquitous in biology, from fur on the bodies of mammals, over trichomes of plants, to the mastigonemes on the flagella of single-celled organisms. While these long and slender protuberances are passive, they are multifunctional and help to mediate interactions with the environment. They provide thermal insulation, sensory information, reversible adhesion, and surface modulation (e.g., superhydrophobicity). This review will present various functions that biological hairs have been discovered to carry out, with the hairs spanning across six orders of magnitude in size, from the millimeter-thick fur of mammals down to the nanometer-thick fibrillar ultrastructures on bateriophages. The hairs are categorized according to their functions, including protection (e.g., thermal regulation and defense), locomotion, feeding, and sensing. By understanding the versatile functions of biological hairs, bio-inspired solutions may be developed across length scales.

## Introduction

Given the bottom-up approach that biology uses to create materials, fibrous structures formed by molecular chains are found everywhere. For example, internally in the form of collagen [1] and microtubules and microfilaments [2], and externally in the form of silk [3] and hair [4–5]. Among these prevalent, quasi-one-dimensional structures, here we loosely define biological “hairs” as high-aspect-ratio structures that are external and passive. This definition is loose yet intuitive. First, a structure must be on the exterior of an organism to be considered as “hair”. This excludes the internal one-dimensional structures such as microfilaments, veins, or bones. Second, in the definition presented here, “hairs” need to be passive, that is, the high-aspect-ratio structures must not be internally active. Obviously, this excludes organisms’ slender body parts, such as elephant trunks, the legs of mammals and insects, and the cilia and flagella of eukaryotic microorganisms. As a side note, flagella of eukaryotic cells (e.g., algae, protists, and sperms) and prokaryotic cells (bacteria) should not be confused. Eukaryotic flagella are essentially the same organelles as cilia, consisting of a well-organized microtubular backbone and orchestrated internal protein motors, whereas bacterial flagella are simply passive, stiff filaments. The passive nature of the hairs does not lessen their importance. They play a crucial role in mediating an organism’s interactions with the environment, serving various functions depending on their deformations, which are driven purely by their surroundings. Altogether, following the definition above, the structures covered in this review include the hair and fur of mammals, the feathers of birds, the trichomes of plants, the setae of arthropods, and the ultrastructures of single-celled organisms.

**Figure 1:**
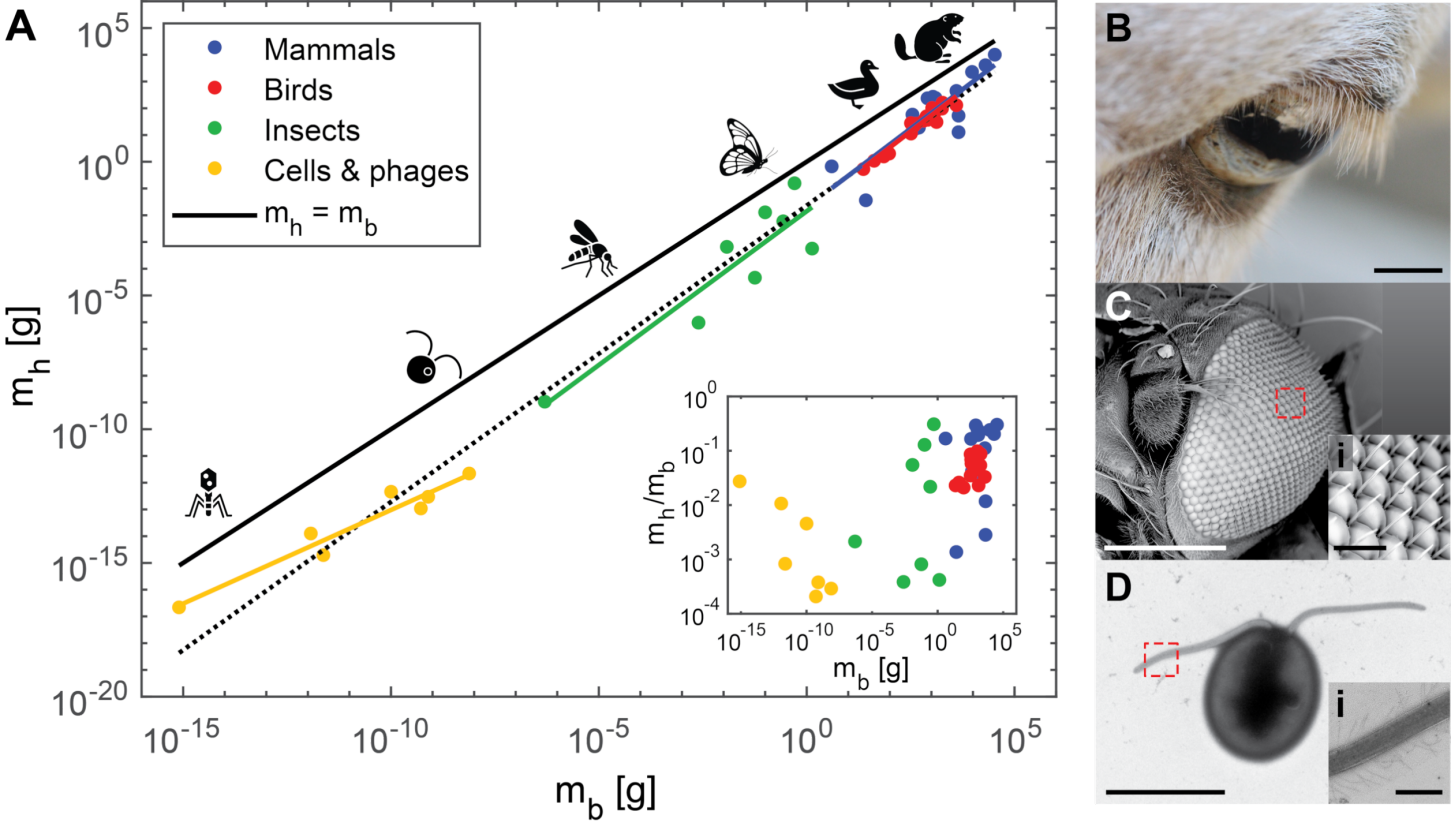
Scaling of hair across body size. (A) Scaling of hair mass *m*_h_ versus body mass *m*_b_. The dots represent data with different colors referring to mammals (blue) [5], birds (red) [9–10], insects (green) [5], and cells and phages (yellow) [11–23]. The dashed black line represents the best power-law fit for all of the data: 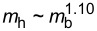 with *R*^2^ = 0.98 and 95% confidence interval (CI): (1.07, 1.15). The solid black line represents equal masses: *m*_h_ = *m*_b_. The solid lines with different colors represent the best power-law fits for the different groups: mammals (blue) 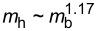 with *R*^2^ = 0.74 and CI: (0.74, 1.61), birds (red) 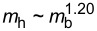 with *R*^2^ = 0.94 and CI: (1.06, 1.34), insects (green) 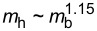 with *R*^2^ = 0.81 and CI: (0.60, 1.70), and cells and phages (yellow) 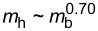 with *R*^2^ = 0.93 and CI: (0.48, 0.92). The inset shows a plot of the ratio of hair mass to body mass *m*_h_/*m*_b_ as a function of body mass *m*_b_. The ratio appears to increase with body mass following a Spearman rank test (ρ = 0.55, *p* = 3.35 × 10^−5^). The silhouettes mentioned below are from Noun Project. They are distributed under the terms of the Creative Commons CC BY 3.0 License, https://creativecommons.org/licenses/by/3.0, and attributed to the following creators: Andre Buand (phage), ProSymbols (mosquito), Creative Stall (butterfly), Laymilk (duck), and Pham Thanh Loc (beaver). (B) The eye of a sheep. Scale bar represents 10 mm. (C) The eye of a fruit fly and (i) close-up of its ommatidia. Scale bars represent 200 µm and (i) 20 µm, respectively. (D) Green microalgae and (i) close-up of its flagellum with mastigonemes. Scale bars represent 10 µm and (i) 1 µm, respectively.

Figure 1A shows how the total hair mass *m*_h_ scales with body mass *m*_b_. For *m*_h_, a material density of 1 g·cm^−3^ was assumed. A relationship slightly exceeding isometry is observed, where 
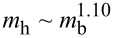
 with 95% confidence interval (CI) of (1.07, 1.15) for the exponent. For purely isometric scaling, if body mass decreases or increases by a factor of 100, then total hair mass decreases or increases by that same factor, respectively. Isometric scaling supports the fact that, with respect to certain characteristics, organisms are scaled copies of each other [6]. For example, as expected from isometry, the total surface area of a salamander was found to scale with 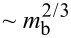 [7], and the same scaling was found for the total area of adhesive pads of animals within the same phylogenetic class, order, family, genus, and species [8].

However, hair mass deviates slightly from isometry, and it appears that larger organisms are more “hairy”. First, the exponent for power-law fits increases with size, as evidenced by comparing the fits for cells and phages, insects, mammals, and birds (see caption of Figure 1A). Second, from the inset of Figure 1A, the ratio of hair mass to body mass *m*_h_/*m*_b_ is higher for larger organisms. A Spearman's rank test supports this observation, with ρ = 0.55, which corresponds to an increasing trend between *m*_h_/*m*_b_ and *m*_b_. Therefore, it seems that larger organisms dedicate more energy and resources to growing and maintaining hair. This finding motivates the following questions: (1) What are the purposes of hair? (2) How do these purposes vary with organism size?

For countless animal species, hairs are strategically placed throughout the body, varying in size and structure. Figure 1B–D show examples of various hairs found in mammals, insects, and micro-algae, respectively. Depending on their location and configuration, hairs serve a multitude of functions that can contribute to an organism’s homeostasis. The diversity of their function is exemplified by hair's resistance to heat transfer in humans [4,24], and the role of hair in sensing mates by male mosquitoes [25]. Additionally, plants may exhibit hair-like fibrillar structures, such as the nanometer-thick mastigonemes on the flagella of microalgae [26] and the high-aspect-ratio, hair-like trichomes on plant surfaces [27]. Overall, to promote homeostasis in plants, animals, bacteria, and bacteriophages, fibrillar structures contribute to the following functions: protection (e.g., thermal insulation and defense), locomotion and feeding, and sensing. This review will present how biological hairs, or fibrillar structures, contribute to those functions across 20 orders of magnitude in organism mass and six orders of magnitude in hair thickness, from the nanometer-thick fibers on bacteriophages to the millimeter-thick hair and fur on mammals.

## Review

### Protection

**Figure 2:**
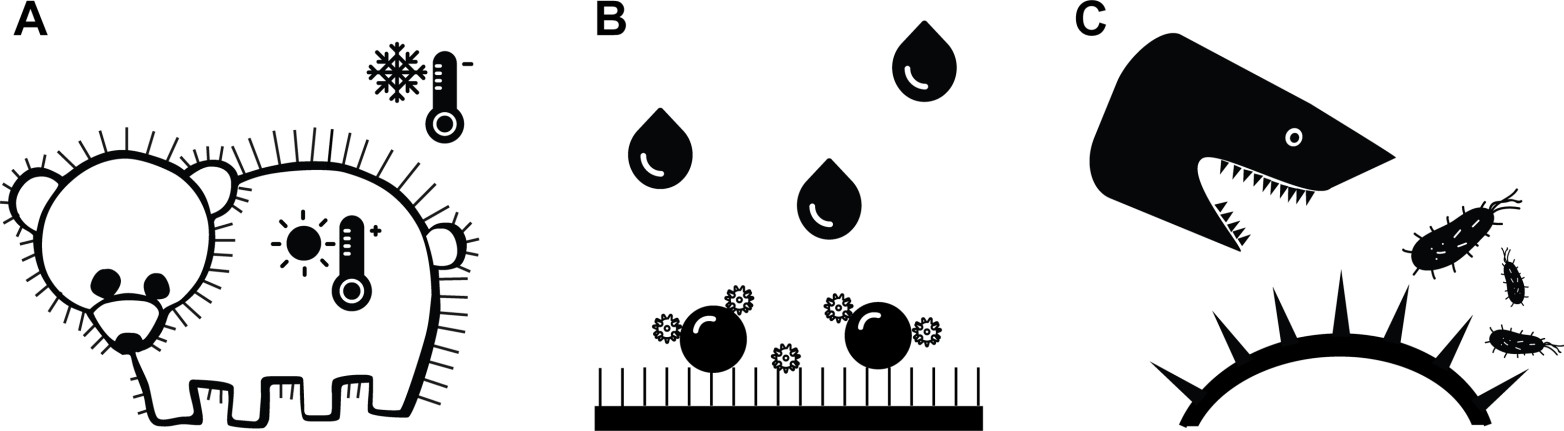
Protection through hairs. Schematics showing (A) an array of hairs providing thermal insulation, (B) a superhydrophobic hairy surface with self-cleaning properties, and (C) hairs defending from unwanted interactions with other organisms. The silhouettes mentioned below are from Noun Project. They are distributed under the terms of the Creative Commons CC BY 3.0 License, https://creativecommons.org/licenses/by/3.0, and attributed to the following creators: madness stock (hot and cold thermometers), Petra Prgomet (bear), Manish (falling water drops), Parallel Digital Studio (pollen grains), and twist.glyph (bacteria).

Plants and animals often encounter potential danger in their surroundings. For example, extreme weather, such as precipitation and low temperatures, predators, and disease vectors. Because of their protruding, fibrillar structures serve as one of the first lines of defense against such dangers. They can protect against heat loss by providing insulation (Figure 2A), prevent the penetration of water through hierarchical superhydrophobicity (Figure 2B), or provide protection from predators or disease vectors through mechanical interactions (Figure 2C).

#### Thermoregulation

Regarding thermal regulation, mammals have evolved certain traits that differentiate them from other animals. In addition to regulating their temperatures by exploiting metabolic processes, mammals tend to be covered in dense coats of fur or hair. A key attribute of these fibrillar structures that promotes insulation is their air-filled center core [28].

Animal pelts and furs are still being utilized by humans as jackets and blankets in order to provide thermal insulation. The fur trade still rears 100 million animals annually, and millions of wild animals are caught in the U.S. every year for their fur [29]. While their continued use is ethically debatable, furs have presumably persisted because of their thermal insulation properties. In mammals, the thickness and packing density of hair arrays was found to coincide with the geometrical parameters that minimize convective heat loss, with the hair diameter *d* scaling as 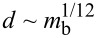
[5,24].

In aquatic mammals, hair morphology, including shape and packing density, differ from terrestrial mammals in order to maintain a trapped air layer within the arrays of hair when submerged in water [30]. Hairs of aquatic animals have been found to be flatter, shorter, and packed in higher densities. Additionally, mammals that also rely on blubber for insulation, such as sea lions and walruses, were found to have lower hair-packing densities [30]. In extreme cases of reliance on blubber (e.g., in bowhead whales) instead of hair for insulation, arrays of hairs are limited to specialized regions where sensory information can be measured, for example, around the chin, lips, and blowhole [31].

While dense arrays of hair can trap a thermally insulating layer of air to protect the animal from cold temperatures, sparse arrays of hair can act like fins, which enhance heat exchange with the surroundings and help to cope with hot temperatures. When hair densities are low, such as in elephants, sparse hair arrays can help to shed heat [32]. The hair density of elephants is around 0.03–0.07 hairs per square centimeter, which is more than three orders of magnitude sparser than the typical hair density on human head (200−300 hairs per square centimeter) [32].

Hairs on humans have also been reported to protect the skin from UVA and UVB radiation from the sun [33]. UV radiation from the sun can not only heat up human skin but is also linked to skin cancers. Therefore, in mammals and birds, hairs provide protection from thermal effects, depending on their density, and from cancer-causing radiation. This demonstrates the multifunctionality of hairs, even within one species, such as humans.

When comparing fur and feathers, it has been found that feathers can outperform fur in protecting against solar radiation. In arid environments in Australia, the feathers of emus (*Dromaius novaehollandiae*) prevent nearly all solar radiation from reaching the bird’s body, while the fur of red kangaroos (*Macropus rufus*) prevents 75–85% of the solar radiation from reaching the mammal’s body [34]. It is thought that the deep coat of feathers protects from solar radiation, so the emus are able to reside in the open without needing to search for shade to cool down. In the ground hornbill (*Bucorvus leadbeateri*), lash-like feathers on the upper rim of their eyelids were found to provide shading from the sun to protect their corneas from intense sunlight [35].

At the scale of insects, setae may also contribute to thermoregulation. Bumblebees, which inhabit globally northern regions, possess dense arrays of setae on their thorax, while other species of bees inhabiting the tropics and hot deserts have very sparse arrays of setae [36]. Such a stark difference is associated with the colder temperatures that bumblebees have to contend with. However, there are trade-offs in possessing dense arrays of fibrillar structures, that is, they contribute to increased aerodynamic drag. Wasps, which are predators that need to outpace their prey during flight, do not possess such insulating arrays of setae [36].

Finally, when an organism is extremely small, such as single-celled organisms and bacteriophages, thermoregulation is limited. Theoretically, the largest temperature difference that a cell with a diameter of 10 µm and calorimetric heat generation of 100 pW can experience is only ≈10^−5^ °C [37]. Additionally, even if a cell of the same size was capable of maintaining a 10-µm-thick air layer (with thermal conductivity of 3 × 10^−3^ W·m^−1^·K^−1^) along its surface, following steady-state one-dimensional heat conduction, it could still only experience a temperature difference of ≈10^−4^ °C. Therefore, thermal insulation would have a negligible effect on thermoregulation at this scale. Instead, cells may be able to regulate their metabolic rates in response to changes in environmental temperatures [38].

#### Wettability

Superhydrophobic surfaces have the unique capability of preventing water from spreading; thus, they exhibit low wettability. In order to achieve superhydrophobicity, surfaces should have structural hierarchy and be composed of materials with low surface energy. The classic example of such a surface in nature is the lotus leaf [39], which possesses wax-covered microscopic pillars. The superhydrophobic surface is self-cleaning since water droplets bead up on the surface, and, when they roll off, they pick up any dirt or other particles and remove them from the leaf’s surface. This phenomenon was termed the “Lotus effect” and has been translated to the development of a self-cleaning paint called Lotusan^®^.

Superhydrophic, fibrillar surfaces are also present in animals, such as insects, spiders, and geckos. Similar to plants, these structures help to maintain a clean body surface by enabling the rolling-off of water, which collects unwanted contaminants, or by providing low adhesion. Such structures are typically found on body parts where contamination is common, such as adhesive pads [40], or where cleanliness is crucial for survival, such as insect wings [41].

Hairs provide more ways to prevent or clean contamination. For a dedicated review on the topic, please see [5]. However, we will mention some of the cleaning functions of hairs here. Hairs around the eyes of mammals (eyelashes) and on the eyes of insects (interommatidial setae) have been found to minimize the deposition of particle-laden contaminants through aerodynamic interactions [42–43]. Hairs on honey bees have been found to facilitate both the collection and removal of pollen grains through the geometries of the hair arrays on their eyes and grooming appendages [44]. Mammalian fur effectively sheds contaminants because the hair deflects when exposed to a fluid flow. This deflection generates a shear flow that removes contaminants [45].

In addition to superhydrophobicity, in certain water plants, such as *Salvinia* spp., specialized structures have been observed to combine superhydrophobicity and superhydrophilicity [46]. In these plants, the fiber-like structures have hydrophilic tips, while the rest of the structure is hydrophobic. The combination of these different wetting properties enables the plants to maintain a stable layer of air while underwater. The hydrophilic tips pin the water surface so that it does not penetrate the fiber array and, thus, trap an air layer directly on the plant’s surface.

While the combination of hydrophilic tips with superhydrophobic structures enables stable air film retention underwater, some animals exploit superhydrophobic surfaces for various functions on or under the water surface. For example, water striders (*Gerridae* spp.) possess superhydrophobic structures on their limbs, which help them locomote on the water surface [47]. Similarly, groups of ants form rafts to float on water and escape flooded regions [48]. This function relies on the wetting properties of their cuticle and its substructures. When underwater, spiders, such as the diving bell spider (*Argyroneta aquatica*), and insects, such as aquatic bugs and beetles, use hydrophobic hairs to trap air and form an air bubble that encompasses their body [49–50]. Insects, such as the green dock beetle (*Gastrophysa viridula*), trap air between the adhesive fibers on their footpads when walking underwater to generate adhesion [51].

#### Mechanical protection

While hairs provide protection via their thermal and chemical properties, they also offer protection based on their mechanical properties. Hairs are typically made of stiff materials, such as keratin and chitin, that have Young’s moduli of the order of gigapascals, comparable to typical values for wood. Therefore, they can be quite robust to mechanical stimuli from external sources.

Mammals possess guard hairs, that are interspersed with the rest of their body hairs or furs. These hairs are distinctly thicker than the rest and have been reported to help provide protection to the rest of the mammal’s coat from abrasion [52]. Guard hairs are also involved in mechano- and thermosensation [53–54]. In addition to guard hairs, some mammals have developed spines or quills to provide protection from predators [52]. These are typically thicker but still made of the protein complex keratin.

Plants also make use of fibrillar structures to provide defense against predators. These structures are known as trichomes and vary in morphology and density. While trichomes may also secrete chemicals to warn predators, they can impale insects and their larvae when they have a hooked morphology or even sting herbivores [55]. It has been observed that plants with higher densities of trichomes suffer less from insect herbivory. Also, there is a reduced incidence of internal egg laying by insects with ovipositors [55].

### Locomotion and feeding

**Figure 3:**
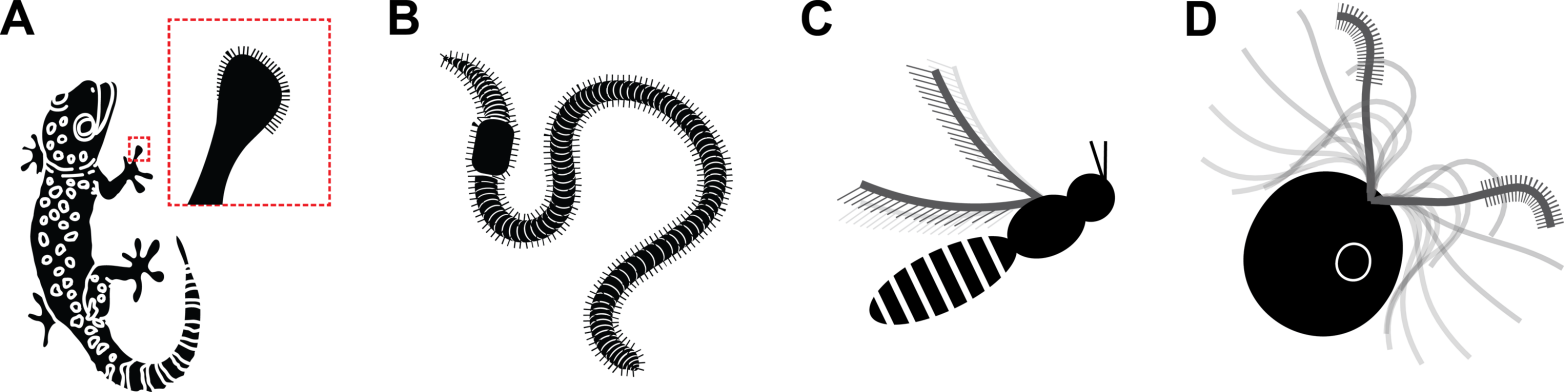
Locomotion enhanced by hairs. Schematics showing (A) an array of hairs (or setae and spatulae) on the toe pad of a climbing gecko, (B) hairs (or setae) on the body of a burrowing earthworm for anchoring when pushing through soil, (C) hairs (or bristles) on the wing of a thrip, instead of a thin membrane as in other flying insects, and (D) hairs (or mastigonemes) on the flagella of swimming microalgae. The silhouettes from (A, B) are from Noun Project. They are distributed under the terms of the Creative Commons CC BY 3.0 License, https://creativecommons.org/licenses/by/3.0, and attributed to the following creators: Hey Rabbit (gecko) and DracoVisions (earthworm).

While hairs can help to protect organisms and to promote homeostasis, strategically placed arrays of hairs are crucial for locomotion through various mediums, such as granular soil, air, and water. By possessing hairs on appendages, organisms across wide length scales are capable of enhancing their locomotory performance. Examples are reversible adhesion in geckos (Figure 3A), anchoring in burrowing earthworms (Figure 3B), flying in bristled-wing insects, such as thrips, (Figure 3C), and swimming in unicellular microorganisms, such as microalgae (Figure 3D).

#### Climbing

Adhesive hairs have been observed on the foot and toe pads of insects, spiders, and geckos [56]. These hairs range in diameter from hundreds of nanometers in geckos, to micrometers in spiders, to tens of micrometers in insects. The hairs are capable of generating adhesive forces through either capillary interactions, when an adhesive fluid is present [57], or intermolecular interactions, when no adhesive fluid is present [58]. In addition to adhesive forces that enable inverted climbing, the hairs can also generate friction forces whenever the animals climb on vertical surfaces [59].

For geckos, these adhesive hairs are referred to as setae, which branch into spatulae at the tips [60]. The branching ensures a high density of hairs to generate high adhesive forces [61]. With advances in fabrication techniques at the micrometer scale, gecko-inspired adhesive hairs have been developed, which are capable of generating adhesion without the use of glues or fluids [62–63].

For insects, the adhesive hairs are around one order of magnitude thicker than those of geckos, and they rely on fluid secreted by the hairs to generate adhesive forces. The hairs on insect footpads can vary in morphology, and these variations have been linked to their functions [57,59]. Green dock beetles (*G. viridula*) have been observed to possess three distinct types of adhesive hairs: discoidal, spatula-shaped, and sharp-tipped. The males possess adhesive hairs with discoidal tips, which are capable of generating large adhesive forces [57]. They are hypothesized to be used by males to attach securely to females when mating [59]. Both males and females possess adhesive hairs with spatula-shaped and sharp tips. The spatula-shaped tips enable reversibility of adhesion, while the sharp-tipped hairs are used for generating friction [59].

For microorganisms, while gravity is less of a concern, adhesive hairs are no less useful than for insects and larger animals. Microalgae, such as *Chlamydomonas reinhardtii* (10 µm in size), possess ≈100 nm long thin hairs on their flagella [11], which help them to attach to surfaces to glide or to attach with other cells to mate. Bacteria, such as *Pseudomonas aeruginosa* (2 µm in size), use thin filaments (up to several micrometers long), known as pilus (type IV), to attach to surfaces and, in effect, “tow” themselves around on the surface [64]. Bacteriophages (≈100 nm in size) rely on their tail filaments to attach to their hosts [21–22].

#### Burrowing

The use of hairs to generate frictional forces is not unique to animals that climb. Hair-like setae on the skin of earthworms aid in burrowing by increasing friction and providing anisotropic anchoring [65–67]. When burrowing, the earthworm mainly uses cavity expansion to create a burrow. It expands some segments of its body to anchor itself, while elongating other segments to push through the soil [68].

This kind of motion is called peristaltic motion since the coordination of expansion and elongation of segments resembles a wave traveling through the worm’s body. It is similar to the motions exhibited by intestines during digestion [69]. The body expansion (increased cross-sectional size) and elongation are controlled by the worm’s circular muscles. When the worm stiffens its circular muscles, the corresponding body segments expand and the setae are erected, helping the worm to anchor tighter to the surrounding soil. Meanwhile, when the circular muscles relax, the setae deflect and interact less with the soil. This anisotropic anchoring has been realized in a bio-inspired burrowing soft robot [70].

#### Flying

Flying organisms span about eight orders of magnitude in mass, ranging from the smallest known parasitoid wasp (*Dicopomorpha echmepterygis*, 2.5 × 10^−8^ kg) to the great bustard (*Otis tarda*, 21 kg). The fluid regimes experienced by these organisms vary greatly with scale, from a highly viscous, laminar environment at the smallest sizes to an inertial, turbulent environment at the largest sizes. Thus, the locomotory appendages of these organisms vary widely with size and the fluid regime they experience.

Structurally and developmentally, feathers are analogous to hair. Bird feathers, like hair, are complex structures made primarily of keratin. Despite approximately 200 million years of independent evolution, feathers and hair follicles share numerous structural similarities, including the presence of a dermal papilla and a dermal sheath [71]. However, unlike hair, feathers also have a dermal pulp, which is essential in growth and regeneration during feather cycling [72]. Much like the mammalian hair cycle [4], feathers are conveniently repaired during grooming and replaced seasonally during molt.

Feathers are highly structured and exhibit self-similarity. They are comprised of a central rachis, which gives rise to barbs. These barbs then branch into barbules, which, in turn, branch into smaller hook-like projections called barbicels. These barbicels cause the barbs to interlock, resulting in a continuous feather surface with relatively low air transmissivity [73]. Many birds have feathers that exhibit lobate cilia and hooked rami, which hook and loop together to prevent gaps between feathers [74].

Beyond forming the aerodynamic surfaces necessary for flight, feathers often exhibit species-specific adaptations. For example, owls have serrations on their leading-edge primary feathers, which are known to reduce noise during flight by mitigating flow instabilities [75–76]. Conversely, many birds use their feathers to produce sound through a variety of mechanisms, including aeroelastic flutter and mechanical rubbing [77–78].

Around one third of birds, notably crepuscular and nocturnal species, such as nighthawks, have facial bristles that resemble mammalian whiskers [79]. These bristles are hypothesized to act as tactile sensors and may aid in prey handling, collision avoidance, foraging, or navigation, as well as provide eye protection [80–81].

Bats are the only mammals capable of powered flight. Their wing membrane is covered with short hairs, which act as tactile airflow sensors [82–83]. The hairs grow sparsely on the membrane of the wing and in fringes on the wing’s leading edge. The neurons associated with these hairs can discriminate airflow directionality, and exhibit the highest firing rate when airflow is reversed, which is associated with slow flight and stall [84]. Indeed, when these hairs are removed, bats alter their flight performance by increasing speed and reducing their turning radius [85].

The membranous wings of insects are covered with bristle (or “hair”) sensilla that act as airflow sensors [86–87]. In Odonata wings, bristle sensilla account for approximately 60% of all wing sensors [88]. In some cases, these bristle sensilla are highly tuned for specific airflow conditions. For example, tests on the silkworm moth (*Bombyx mori*) revealed that their bristle sensilla responded to vibrating air currents but not to constant flow [89]. It is hypothesized that the height of these bristles matches the height of the boundary layer, but further aerodynamic testing is necessary [88].

The smallest insects, such as beetles, thrips, and parasitoid wasps, possess wings made entirely of bristles [90–96]. The bristles (or setae) of these wings support flapping flight by reducing inertia, enhancing aerodynamic performance, and facilitating their deployment (i.e., folding and unfolding). The wing acts as a leaky paddle, and can produce 66–96% of the aerodynamic drag force of an equivalent membranous wing [94]. Conventionally, the competition between inertial and viscous forces is captured by the Reynolds number (Re), and a large Re indicates a dominant role of inertia. At Re ≈ 4–60, the effects of viscosity are significant, and inertial forces are relatively weak [97–98]. Consequently, traditional steady-state lift-based flight, as observed in larger organisms, is not possible. Thus, miniature insects use unsteady aerodynamics through a combination of wing flapping, wing clap-and-fling, and recapture of vortices to generate lift and thrust through the manipulation of air resistance (drag) [99]. In essence, the very smallest insects move by rowing through the air, generating drag much like a paddle. The bristles are also known to improve control of the boundary layer and delay stall via the generation of leading-edge vortices.

#### Swimming

For microorganisms, whose body sizes typically range from 10^−7^ to 10^−4^ m and who live predominantly in water, “inertia is totally irrelevant” [100]. While Re is around 10^5^ for a flying eagle or a swimming whale, it is 10^−3^ to 10^−5^ for moving microbes. A thought experiment gives a straightforward illustration to such drastic distinction [101]. Imagine that an animal flying or swimming at high speed suddenly freezes the motion of its body parts (wings, fins, or flukes), how long would it continue to travel through the medium? While displacement for an eagle or a whale can continue for some time and distance, typically, an *Escherichia coli* bacterium (3 µm long, swimming at 10 µm·s^−1^) will stop immediately, that is, within 10^−6^ s and 10^−10^ m [100].

In this viscosity-dominated regime, because there is no inertia to depend on, microorganisms must constantly deform body parts in a non-time-reversible fashion to swim. Therefore, swimming efficiency depends on the order (or pattern) in which deformations take place. Three types of patterns are the most common: (1) rotation of a corkscrew-like tail found in archaea [102–103] and bacteria [15,104], (2) travelling waves along filaments (flagella) adopted by sperm cells and some algae [15], and (3) cyclic beating pattern consisting of a power stroke of large amplitude and a recovery stroke of small amplitude (similar to the arm movement during breaststroke swimming), which is adopted by microalgae [105] and ciliates [15].

In these locomotory patterns, microbial hairs are consistently involved in drag force generation. The flagella of archaea and bacteria are themselves passive hairs and are driven by protein motors at the base. Hair-like ultrastructures, or mastigonemes, on eukaryotic flagella/cilia comprise helical glycoproteins (≈10–20 nm thick) and lack a membrane [106]. They can be either stiff or flexible. Flagella with thick and stiff hairs (tubular mastigonemes) are sometimes referred to as the “tinsel” type [12,107]. These stiff hairs help to increase the effective surface area of flagella and, thus, enhance swimming speed [15]. Moreover, the stiff hairs help to reverse the resultant swimming direction when travelling waves patterns are employed [108]. For example, the smooth flagellum of sperm [109] and the tinsel-like flagellum of golden algae *Ochromonas*[12] beat in the same pattern, featuring waves travelling away from the cell body. In *Ochromonas*, this results in a swimming direction towards the waves’ travelling direction, while sperm cells swim towards the opposite direction. This modulation effect has already inspired designs of swimming microrobots [110].

The role of thin and flexible hairs (fibrous mastigonemes) is still, to some extent, enigmatic. These soft hairs may appear in a range of number densities, from ca. 1 per micrometer of length of flagellum in *Phytophthora*[107] and *Ochromonas*[12], over ca. 10 per micrometer in the green algae *C. reinhardtii*[26], to 10^2^–10^3^ per micrometer in *Euglena*[111]. At least for the hair density found in *C. reinhardtii*, they do not help the cell to swim faster [26]. Nevertheless, without these hairs, swimming in *C. reinhardtii* is interrupted by frequent and sudden turns [26]. A possible explanation for this is that the fibrous hairs are involved in sensory functions, which may be crucial for stable, controlled swimming [112].

Microbial hairs commonly serve multiple roles at the same time. Hence, one should avoid understanding these hairs’ existence from a single, locomotory perspective. While the flagella of *E. coli* is most obviously an apparatus for swimming motility, it can also help the cell to attach to a surface and act as a sensor thereafter [113]. Intriguingly, even after attachment, having motile flagella still matters for the cell as it appears to enable sensing of substrate stiffness [114]. In addition to flagella, other hairs of E. coli include the type-I pili (frimbriae) and type-IV pili [113]. Collaboration between these hairs also helps the cell. When pproaching a solid surface, the cells become trapped as they move in circular orbits because of hydrodynamic effects [101,104]. While staying close to the surface may be beneficial as it facilitates surface attachment and, hence, the formation of bacterial biofilms, remaining in circular trajectories hinders the cell’s ability to explore the surface thoroughly. Thus, possibly with the help of the other hairs, *E. coli* near the surface can transiently attach to the surface to break the circular trajectories, thus, pushing their exploration efficiency close to the theoretical optimum [115].

Developing tools with one-dimensional structures is arguably the most basic and economical (materials-wise) solution for microorganisms. In this light, the “hairs” are their available tools, where most tools happen to look alike. This is the primary reason why microbial hairs defy easy classifications. Future research linking form and function in microbial hairs may lead to a better understanding of their evolution, as well as providing inspiration for the development of functional fibrillar structures at the micrometer and nanometer scales.

#### Filter feeding

Locomotion is key for searching for food, and hairs may also serve crucial roles in feeding, particularly via filtering. Filter feeding uses a porous material to capture prey and nutrients in fluid flows. Dense arrays of hairs may serve as the porous material that captures the food, separating it from the surrounding flow or from other unwanted objects. The capturing can occur via sieving, where food larger than the gaps between the fibers gets trapped, or through hydrodynamic interactions that transport food to the fiber surface, where it can stick and become trapped [116].

At the largest scales, baleen whales (Mysticeti) use keratinous fibers, or baleen, in their mouths instead of teeth to filter and capture prey [117–119]. When feeding, the whales use three different strategies, depending on their species. Bowhead and right whales (Balaenidae) use ram filter feeding where they continuously swim through groups of prey with their baleen exposed, collecting prey while the filtered water exits through an opening in the posterior of their mouths. Rorqual whales (Balaenopteridae) use lunge feeding where they swallow mouthfuls of prey and water and then push the seawater out through their bristled baleen in order to isolate the prey for swallowing. Grey whales (*Eschrichtius robustus*) use suction filter feeding [119].

At the smaller scales, aquatic insects of the orders Ephemeroptera, Trichopteram, and Diptera use filter feeding to consume organic matter from their aqueous environment [120]. The fibrillar filters used by insects include setae, mouth brushes, and fans. The setae are present around the mouthparts or forelimbs and may be lined with arrays of smaller fibers, called microtrichia. The mouth brushes are dense arrays of fibers present on the lower jaw, or labrum. Fans are arrays of fibers that can be opened (splayed) and closed. The captured organic matter in the fans is consumed by sweeping the mandibles over the closed fans [120].

Choanoflagellates are unicellular organisms that use filter feeding. They drive fluid flow through a conical filter consisting of microvilli with diameters of 100–200 nm, spaced 200–700 nm apart [121–122]. While the microvilli contain actin and myosin, which together enable motility during escapes and help to transport trapped organic matter for consumption [123], they function passively when filtering organic matter. The structure driving the fluid flow through the filter remains elusive. A flagella alone does not seem to provide enough flow to explain the experimentally observed filtering rates. However, it has been proposed that a flagellar vane, which behaves like an undulating wall, could induce enough flow through the conical filter [122].

### Sensing

**Figure 4:**
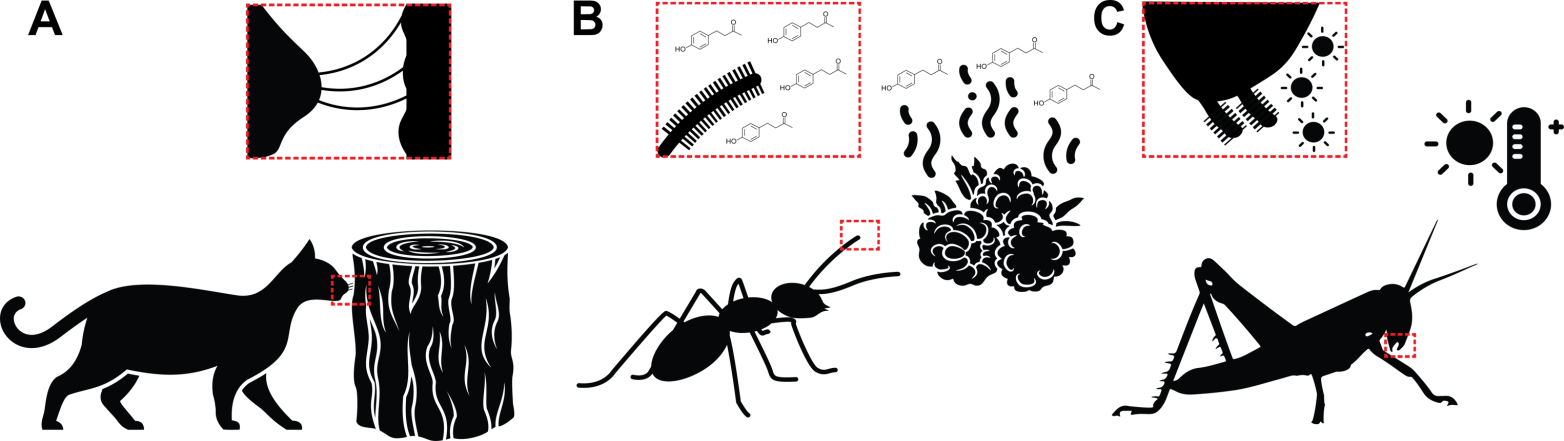
Sensing via hairs. Schematics showing (A) whiskers on cats sensing objects through mechanical interactions, (B) hairs on the antennae of ants detecting the odor of molecules, and (C) hairs on the labial palps of cave-dwelling crickets sensing temperature. The silhouettes mentioned below are from Noun Project. They are distributed under the terms of the Creative Commons CC BY 3.0 License, https://creativecommons.org/licenses/by/3.0, and attributed to the following creators: Hafid Firman Syarif (cat), Fardan (tree trunk), Jacob Eckert (ant), Hey Rabbit (raspberries), bsd studio (odor lines), Ed Harrison (cricket), and madness stock (hot thermometer).

Perceiving the environment using sensory organs in order to respond to stimuli is vital for survival in animals [124]. Hairy receptors are a type of sensing organ that exists widely across nature. They are systematically distributed throughout the surface of the bodies of organisms and play an important role in reacting to external stimuli in order to perceive the environment, such as external touch (Figure 4A), odor (Figure 4B), temperature (Figure 4C), and humidity [125–127]. Hairy receptors can be classified into several types according to their various functions and sensing modes, such as mechanoreceptors, chemoreceptors, thermoreceptors, and hygroreceptors. While there are different types of hairy receptors, depending on their location and type of stimulus they sense, they all generate electrical signals through their sensory cells and transmit the signals to the nervous system in order to paint a picture of the outside world or determine body or appendage orientation [128].

#### Mechanosensation

Hairs, as mechanical receptors, are capable of perceiving and distinguishing a multitude of external stimuli such as touch, vibration, or fluid flows [129–130]. The mechanosensation of hairs relies on the sensory cells at their base. When the hair is deflected by mechanical forces, the membrane potential of the sensory cells is altered, and an electrical signal is sent to the nervous system. By receiving, analyzing, and finally reacting to the signal, the organism is able to respond to changes in the surroundings [131].

Mechanical perception via hair is important for living organisms across length scales and evolutionary backgrounds. Cats, for instance, can sense the position, shape, and texture of objects by moving their whiskers, and can even use whiskers to sense the direction and speed of air flow to help them move in the dark or catch prey [132]. A review of hairy sensation in mammals can be found here [133]. Spider appendages [134], cockroach antennae [135], and cricket cerci [136] possess hairs capable of detecting delicate vibrations, airflow, and interactions with various objects, enabling them to locate their prey, evade obstacles, or detect potential dangers [130,137]. Airflow sensors with bio-inspired, fibrillar structures based on the working principles of cricket cerci, which, when clustered in arrays, aid in detection of oscillating flows following “viscous coupling” [138], have been developed [139].

At the microscopic scale, microalgae, such as *C. reinhardtii*, may utilize the hair-like ultrastructures, or mastigonemes, on their flagella to sense fluid flow while swimming [26]. The mastigonemes have been observed to be anchored to a channel protein that shows ion conductivity, and the mastigoneme–channel protein complex may provide mechanical gating to sense deflections of the mastigonemes caused by fluid flow [112]. Additionally, for bacteria, *E. coli*, their passive flagella have been linked to sensing the material stiffness of surfaces they attach to [114].

Clusters of hairs, or hair plates, on the limbs of insects are used for proprioreception or to sense the orientation and motion of the limbs, which helps in their control of locomotion [131]. Furthermore, many insects, such as bees, can enhance their foraging speed by utilizing hairy mechanical receptors to detect physical characteristics such as food viscosity and texture [140–141]. Mechanosensing with hairs, regardless of stimulus, relies on deflections of the hairs triggering deformations to the sensory cells they are attached to.

#### Chemoreception

In addition to touch, hairs are able to sense their less-immediate environment by detecting the presence and alteration of chemicals [142], which differs from the way hairs sense touch and vibration. The binding of receptor proteins on sensory cells to chemicals in the air or solution initiates a sequence of biochemical reactions, resulting in the production of electrical signals, which are then transmitted to the nervous system. The brain interprets these electrical signals as a specific scent or flavor after they are processed by the nervous system [143].

Arthropods, including spiders [144], ants [145], and bees [146], possess chemical receptors on their limbs and antennae that detect chemicals in their surroundings, enabling them to locate sustenance, recognize their species, and avoid danger [147]. Moth antennae possess dense arrays of hairs, which have been found to interact with surrounding airflow in order to enhance diffusion of chemicals to the antennae for detection [148]. Based on this knowledge, bio-inspired, fibrillar chemical sensors have been developed [149–150]. Furthermore, insects can utilize hairs to sense atmospheric carbon dioxide [151–152]. The human scalp follicles also possess an olfactory perception and can even stimulate hair growth upon exposure to a specific fragrance [153].

#### Thermosensation

Hair can also act as a temperature sensor, helping organisms to choose the right temperature environment to keep their body thermally stable. The receptors typically have a short hair that protrudes through a small hole to interface with the environment, also known as a peg-in-pit sensillum [154]. The protruding hair-like receptors help to absorb thermal radiation, since the penetration depth of infrared radiation into insect cuticle is quite shallow [155]. Additionally, the hair-like sensillum possesses electron-dense filaments that may improve absorption [154]. Leaf-cutting ants (*Atta vollenweideri*), for instance, can utilize the temperature receptors of their appendages to detect intense heat outside their nests as indicators of where to locate food [156]. Cave-dwelling crickets (*Tachycines plumiopedella*) rely on thermosensation to detect temperature gradients and locate appropriate habitats in the environment, utilizing hair receptors on their labial palps [157]. In plants such as the Venus flytrap (*Dionaea muscipula*), heat was observed to trigger closure of their trap [158]. At the base of their trigger hairs, there are sensory cells that may be triggered by either mechanical or thermal energy [158].

#### Hygrosensation

Studies have also shown that hairs exhibit heightened sensitivity to changes in humidity levels, enabling arthropods to discern variations in air humidity with remarkable precision. There are three potential mechanisms for hygrosensation with hairs or sensilla: (1) Changes in humidity may cause changes in the volume of the sensilla, which could mechanically trigger sensory cells. (2) For hollow sensilla, the external humidity could cause lymph fluid to evaporate, and the change in fluid volume may trigger sensory cells. (3) Changes in humidity could cause changes in temperature of the sensilla and trigger thermoresponsive sensory cells [159]. These sensilla are distributed across the body, including antennae, legs, and other appendages. Insects such as locusts [160] and beetles [161] utilize hygroreceptors on their antennae to detect humidity fluctuations in their environment. Similarly, arachnids such as the harvestman (*Heteromitobates discolor*) also possess hygroreceptive sensilla on their legs [162].

## Data Availability

Data sharing is not applicable as no new data was generated or analyzed in this study.
